# Safety and Efficacy of COVID-19 Vaccines: A Systematic Review and Meta-Analysis of Different Vaccines at Phase 3

**DOI:** 10.3390/vaccines9090989

**Published:** 2021-09-04

**Authors:** Yu-Jing Fan, Kwok-Hung Chan, Ivan Fan-Ngai Hung

**Affiliations:** 1 Department of Medicine, Li Ka Shing Faculty of Medicine, The University of Hong Kong, Hong Kong, China; u3571448@connect.hku.hk; 2 Department of Microbiology, Li Ka Shing Faculty of Medicine, The University of Hong Kong, Hong Kong, China; chankh2@hku.hk; 3 Department of Medicine, Queen Mary Hospital, The University of Hong Kong, Hong Kong, China

**Keywords:** mRNA vaccines, inactivated vaccines, non-replicating vaccines

## Abstract

This systematic review and meta-analysis was conducted to compare the safety and efficacy of 2019 novel coronavirus disease (COVID-19) vaccines according to vaccine platform and severe acute respiratory syndrome coronavirus 2 (SARS-COV-2) infection severity. Articles published between 24 January 2020 and 30 May 2021 were retrieved via a PubMed and EMBASE search. A total of 12 reports on phase-3 clinical trials and observational studies of COVID-19 vaccines were included in the review. In terms of vaccine safety, mRNA vaccines showed more relevance to serious adverse events than viral vector and inactivated vaccines, but no solid evidence indicated that COVID-19 vaccines directly caused serious adverse events. Serious metabolic, musculoskeletal, immune-system, and renal disorders were more common among inactivated vaccine recipients, and serious gastrointestinal complications and infections were more common among viral vector and inactivated vaccine recipients. The occurrence of serious vessel disorders was more frequent in mRNA vaccines. In terms of efficacy, two mRNA vaccine doses conferred a lesser risk of SARS-COV-2 infection (odds ratio: 0.05; 95% confidence interval: 0.02–0.13) than did vaccination with viral vector and inactivated vaccines. All vaccines protected more against symptomatic than asymptomatic cases (risk ratio, 0.11 vs. 0.34), but reduced the risk of severe SARS-COV-2 infection. The COVID-19 vaccines assessed in this study are sufficiently safe and effective. The results indicate that two mRNA vaccine doses prevent SARS-COV-2 infection most effectively, but further research is needed due to the high degree of heterogeneity among studies in this sample. Interventions should be implemented continuously to reduce the risks of infection after one vaccine dose and asymptomatic infection.

## 1. Introduction

The 2019 coronavirus disease (COVID-19) was first identified in Wuhan, China, in December of that year. COVID-19 is caused by a novel coronavirus [severe acute respiratory syndrome coronavirus 2 (SARS-COV-2)], similar to the other two coronaviruses—SARS-CoV and MERS-CoV [1]. Common manifestations of COVID-19 include cough, fever or chills, and shortness of breath, making these diseases difficult to distinguish from influenza [2]. Compared with SARS, MERS and influenza, COVID-19 is transmitted more easily from human to human through droplets and has a higher infection rate, but a lower mortality rate [1]. Given these properties, it spread from its origin to other regions and countries at unexpected speed, causing a global pandemic. Currently, more than 100 million people have been infected globally, and about 2.9 million deaths have been registered globally; this disease has posed an enormous challenge to health systems worldwide [3]. Infection control measures, such as wearing masks, the suspension of public gatherings, school closures, and travel restrictions, remain the mainstays of prevention, although they have seriously impacted daily life and the global economy [4]. Because a standard therapy for COVID-19 is not yet available, effective vaccines are needed urgently to end the global pandemic.

More than 90 vaccines against SARS-COV-2 are being evaluated in clinical trials, and several have been approved for large-scale vaccination, [e.g., CoronaVac (Sinovac, Beijing, China), BNT162b2 (BioNTech, Mainz, Germany), and AZD1222 (AstraZeneca, Cambridge, UK)]. CoronaVac (Sinovac, Beijing, China) and BBIBP-CorV (Sinopharm, Beijing, China) were the first inactivated vaccines developed following the COVID-19 outbreak [5,6]. Such vaccines employ the most traditional platform, by which the virus is inactivated using a physical or chemical method but retains its immunogenicity. When the inactivated virus enters the human body, it induces an immune response but has no pathogenicity. This vaccine technology is considered to be the safest and has been proven to prevent infectious diseases such as influenza and polio [7]. It is also convenient to develop, and thus tends to be used to contain emerging infectious diseases. CoronaVac (Sinovac, Beijing, China) and BBIBP-CorV (Sinopharm, Beijing, China) were developed in China, and their phase-3 clinical trials were performed in various countries, including Brazil, Indonesia, Turkey, and Peru. Data from these trials were published recently for BBIBP-CorV (Sinopharm, Beijing, China) and are under peer review for CoronaVac (Sinovac, Beijing, China) [8]. These two vaccines have been approved for emergency use in many countries and regions, including the United Arab Emirates (UAE), Indonesia, Hong Kong and Macau.

Viral vector vaccines are developed using another important platform. The major COVID-19 vaccines of this type at present [Gam-COVID-Vac (Gamaleya, Moscow, Russia), AZD1222 (AstraZeneca, Cambridge, UK), and CanSino (CanSino Biological Inc., Tianjin, China)] are based on vectors derived from an adenovirus and the recombined spike gene of SARS-COV-2; they stimulate cellular and humoral immunity, generating a more durable and effective immune response than inactivated vaccines [9]. Thus, these adenoviral vector vaccines are being used for emergency prophylaxis during the COVID-19 pandemic [10]. AZD1222 (AstraZeneca, Cambridge, UK), which employs a chimpanzee adenovirus vector, has been used extensively in various countries and regions. Ad26.CoV2.S (Johnson & Johnson, New Brunswick, NJ, USA) and CanSino (CanSino Biological Inc., Tianjin, China), which employ adenovirus serotype 26 (Ad26) and adenovirus serotype 5 (Ad5) vectors, respectively, are single-dose vaccines, and the Russian-manufactured Gam-COVID-Vac (Gamaleya, Moscow, Russia) employs both of these vectors in doses administered sequentially at a 21 day interval. Despite the World Health Organization’s (WHO’s) approval of Ad26.CoV2.S (Johnson & Johnson, USA) and AZD1222 (AstraZeneca, Cambridge, UK) for emergency use, rare cases of thrombosis after the receipt of these vaccines have raised public concern, regardless of the lack of establishment of a clear causal relationship between adenoviral vector vaccine receipt and thrombosis events. 

mRNA-1273 (Moderna, Cambridge, MA, USA) and BNT162b2 (BioNTech, Mainz, Germany) were developed using a platform based on mRNA encoding of the viral protein. The encoded mRNA fragment will be injected into human bodies, and can be translated into antigen proteins in human cells and induce an immune response in the human system [11]. mRNA vaccines can be developed more rapidly than can those based on other platforms; BNT162b2 (BioNTech, Mainz, Germany) was the first COVID-19 vaccine approved for emergency use by the WHO on 31 December 2020, followed by mRNA-1273 (Moderna, Cambridge, MA, USA) on 30 April 2021. mRNA vaccines have been shown to provide enough protection (efficacy > 50%) [12], but the technology with which they were developed is new; it has not been used previously for the mass production of vaccines or prevention of infectious diseases. Thus, long-term surveillance of these vaccines’ safety and efficacy is required. In addition, the storage conditions required for these vaccines due to the instability of the mRNA structure [e.g., −70 °C for BNT162b (BioNTech, Mainz, Germany) and −25 °C for mRNA-1273 (Moderna, Cambridge, MA, USA)] makes them difficult to transport.

About 30 protein subunit COVID-19 vaccines have advanced into clinical trials globally. This platform has been applied successfully in Hepatitis B vaccines. The targeting virus antigen, such as the SARS-COV-2 spike (S) protein or receptor-binding domain (RBD), is manufactured via genetic engineering, and expressed in cells in vitro. When injected into the human body, it elicits the production of corresponding antibodies directly and rapidly [13]. Because these vaccines do not contain a live virus, they are considered to be safe and effective. Phase-1 and -2 results have been reported for NVX-CoV2373 (Novavax, USA) and ZF2001 (Longcom, China), COVID-19 vaccines of this type, and phase-3 clinical trials for them are underway [14,15]. Preliminary analyses indicated that NVX-CoV2373 (Novavax, USA) induces high antibody titers against the SARS-COV-2 S protein, blocking attachment of the virus to human angiotensin-converting enzyme 2, and that ZF2001 (Longcom, China), which targets the RBD of the SARS-COV-2 S protein, effectively blocks receptor binding.

Clear evidence to guide the choice of appropriate vaccines among the variety of candidates for population-based vaccination in different locations is lacking. This systematic review and meta-analysis of the safety and efficacy of vaccines developed using different platforms was conducted to provide more evidence to optimize COVID-19 vaccine usage. 

## 2. Materials and Methods

### 2.1. Search Strategy

To explore the efficacy and safety of available COVID-19 vaccines for which phase-3 trial data were published as of 30 May 2021, the PubMed database and EMBASE were searched with no restriction on language or year of publication, in accordance with the PRISMA guidelines [16] (Table 1). The keywords used for the PubMed search were “COVID-19 vaccine*” AND [(Efficacy OR Effectiveness) OR Safety], and the subheadings used for the EMBASE search were (exp SARS-COV-2 vaccine/or COVID-19 vaccine.mp.) AND [(Efficacy.mp. OR Effectiveness.mp.) OR (Safety.mp. or exp Safety/)]. Additionally, a systematic electronic search of ongoing studies and clinical trials was conducted using the COVID-19 section of the ClinicalTrials.gov database.

### 2.2. Selection Criteria

#### 2.2.1. Inclusion Criteria

The systematic review included studies reporting on SARS-COV-2 vaccines for which phase-3 clinical trial data were available, regardless of vaccine platforms, route of administration and doses. Only full-text reports on randomized clinical trials and observational studies of the efficacy and safety of the vaccines were included, to ensure that a sufficient amount of evidence was obtained for evaluation. Selected articles provided data on the incidence of serious adverse events and cases of (post-vaccination) SARS-COV-2 infection in vaccine and control groups. No restriction on the region in which the trial was undertaken, population examined, or virus variants included was imposed (Table 1).

#### 2.2.2. Exclusion Criteria

Review articles, editorial papers, letters, and preprints were excluded from the review. Reports on animal models, in vitro studies, study protocols, preclinical trials, and phase-1 and -2 clinical trials were also excluded.

### 2.3. Data Extraction and Publication Quality Assessment

Publication details (including the authors and year of publication) and data on the study design, vaccine name, platform, number of doses, population, route of administration, number of people experiencing serious adverse events, number of (symptomatic and asymptomatic) COVID-19 cases, and the severity of SARS-COV-2 infections in vaccine and control groups were collected from eligible publications. Using the “Quality in prognostic Studies (QUIPS) tool” [17], the quality of each included study was assessed in the following domains: study population, study attrition, prognostic factor measurement, outcome measurement, study confounding, and statistical analysis and reporting. Given that a small number of eligible articles had been published at the time that this systematic review was conducted, publication bias was examined as described in Section 2.4.

### 2.4. Data Analysis

Currently, symptoms such as fever, headache, fatigue, and vomiting are reported very commonly by vaccine recipients. The mild and moderate adverse reactions occurring in most cases are not life threatening; some serious adverse reactions, however, require hospitalization and are potentially fatal [18]. Thus, the occurrence of serious adverse events is a very important indicator in the evaluation of vaccine safety. Pooled estimates of odds ratios (ORs) based on the number of study participants experiencing serious adverse reactions and the proportions of serious adverse events (by system organ class) occurring after the receipt of at least one vaccine dose were used to evaluate vaccine safety. Vaccine efficacy was evaluated using pooled risk ratios (RRs) for the numbers of COVID-19 cases occurring after one and two vaccine doses, the numbers of symptomatic and asymptomatic cases occurring after two vaccine doses, and the severity of SARS-COV-2 infections occurring after full vaccination. Meta-analysis of vaccine safety and efficacy were conducted separately, and analysis of subgroups defined according to the vaccine platform and case type were performed to characterize diversity among studies. Vaccine efficacy was determined using the formula (1 − RR) × 100%.

Heterogeneity among studies was evaluated using the Higgins statistic (*I^2^*), with sequential omission of individual studies performed to identify the source of the heterogeneity. When *I*^2^ values were >50%, a random effects model was applied to compute the overall results; otherwise, a fixed effects model was used. Begg’s funnel plots were used to examine publication bias. Asymmetrical plots were constructed to reflect the potential existence of such bias and were adjusted using the trim-and-fill method. All data analyses were performed using Review Manager 5.4 and R 4.0.3 software.

## 3. Results

### 3.1. Study Selection

**Figure 1:**
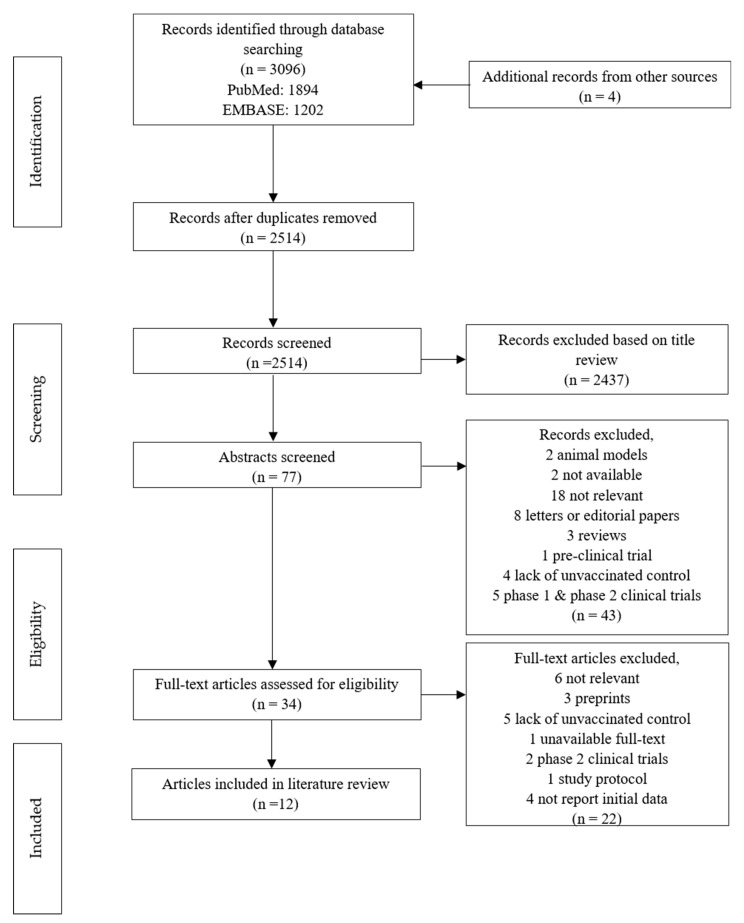
Flowchart for the process and results of study selection in the systematic review.

In the initial literature search, 3100 potential articles were identified up to 30 May 2021 (1894 in PubMed, 1202 in EMBASE and four in the ClinicalTrials.gov database). After the removal of 586 duplicate articles, the titles and abstracts of the remaining 2514 articles were screened, and 2480 articles were excluded based on the inclusion and exclusion criteria. The assessment of the full texts of the remaining articles led to the inclusion of 12 articles describing eight clinical trials and four observational studies in the literature review (Table 2). A flowchart of the literature search is provided as Figure 1.

### 3.2. Description of Studies

Clinical trial data was reported for 19 vaccines as of 30 May 2021, but phase-3 trial results had been published for only seven vaccines: mRNA1273 (Moderna), BNT162b2 (BioNTech), AZD1222 (AstraZeneca), Gam-COVID-Vac (Gamaleya), Ad26.CoV2.S (Johnson & Johnson), BBIBP-CorV (Sinopharm) and an inactivated vaccine developed from WIV04 (Sinopharm). Six of the included articles described studies of mRNA vaccines [19,22,25,26,27,28], five articles reported on studies of non-replicating viral vector vaccines [20,21,23,24,29], and one article described a study of two inactivated vaccines [29]. All vaccines reported on are administered by intramuscular injection. Relative safety statistics were provided in six articles [19,20,21,22,24,30]; protection against SARS-COV-2 infection was evaluated by comparing the incidence of SARS-COV-2 infections in vaccine and control arms in all 12 studies [19,20,21,22,23,24,25,26,27,28,29,30]. Vaccine efficacy after full vaccination was evaluated in all 12 studies; cases were classified as symptomatic and asymptomatic in 10 articles [19,21,22,23,24,25,26,28,29,30]; intervals between the first and second vaccine doses were reported in six articles [19,21,22,26,27,29]; and the severity of post-vaccination SARS-COV-2 infection was reported in seven articles [19,20,21,22,25,26,30]. Synthesized data from phase-3 clinical trials of AZD1222 conducted in more than one country were reported in three articles [20,23,29], and the efficacy of this vaccine against B.1.1.7 and non–B.1.1.7 lineages of SARS-COV-2 was reported in one article [23].

The included studies were conducted in different countries and regions with at least 8000 participants each, and dropout rates were very low. In addition, most studies were undertaken with high-risk populations, such as healthcare workers. Generally, vaccination required two doses administered at a 28 or 21 day interval, and all participants were monitored for some time to evaluate vaccine safety and efficacy. Table 2 summarizes information extracted from the selected literature.

### 3.3. Meta-Analysis Results

#### 3.3.1. Vaccine Safety

Meta-analyses of vaccine safety were conducted in six studies, with eight comparisons of ORs, because Baden et al. [19] evaluated the occurrence of serious adverse events after one and two vaccine doses, and Sinopharm produced two inactivated vaccines developed from the SARS-COV-2 WIV04 and HB02 strains. BBIBP-CorV, which was developed from the HB02 strain, in collaboration with the Beijing Institute of Biological Products, has been listed as an emergency-use COVID-19 vaccine [30]. All adverse events occurring after vaccination in the included studies were reported using Medical Dictionary for Regulatory Activities (MedDRA) terms [18]; and serious adverse events (hospitalization, death, and other life-threatening events) were classified using the US Food and Drug Administration’s Toxicity Grading Scale [31]. The risk of serious adverse event occurrence after at least one dose was greater for mRNA vaccines [OR = 1.47; 95% confidence interval (CI), 0.65–3.29] than for non-replicating viral vector (OR = 0.76; 95% CI, 0.62–0.93) and inactivated (OR = 0.79, 95% CI, 0.62–1.00) vaccines (Table 3), but the 95% CIs indicate the lack of clear evidence for a direct relationship between the receipt of any of these vaccines and serious adverse reactions. Although no significant association between serious adverse events and vaccines was observed in the studies, with the exception of that of Baden et al., which reported a significant association between the receipt of the second vaccine dose and the occurrence of serious adverse events (OR = 4.63; 95% CI, 1.33–16.13) [19], obvious heterogeneity among the three vaccine subgroups was observed (*I*^2^ = mRNA vaccines 61% vs. viral vector vaccines 0% vs. inactivated vaccines 0%; Figure S1). Most heterogeneity derived from the mRNA vaccine subgroup. Thus, a sensitivity analysis was conducted using the sequential omission approach. The omission of data from Baden et al. [19] reduced the degree of heterogeneity to 23.8%, which can be regarded as no significant heterogeneity in other studies. Hence, it was indicated that there was no significant association between serious adverse events and COVID-19 vaccines. 

Serious adverse events in all system organ classes were rare (Table 4). Serious metabolic (19%; 14%), musculoskeletal (9%; 13%), immune-system (8%; 6%) and renal disorders (17%; 8%) were more common among recipients of the two inactivated vaccines, and serious gastrointestinal tissues and infections were reported more commonly among recipients of the viral vector and inactivated vaccines (9–34%), which were less seen in mRNA vaccines (<5%). Serious vessel or lymphatic disorders, such as thrombosis or lymphadenitis, were more easily observed in mRNA vaccines, although these disorders also occurred in the two other vaccine platforms. 

#### 3.3.2. Vaccine Efficacy

Data from six and 10 publications were used to estimate vaccine efficacy after one and two doses, respectively, with random-effects models used to pool RRs, because >50% heterogeneity among studies was detected (Figures S2–S4). The overall RR for SARS-COV-2 infection after two vaccine doses was 0.17 (95% CI, 0.07–0.40; Table 3), indicating that the vaccines examined in the included studies are effective. The RR for SARS-COV-2 infection was lower by about 0.3 for the mRNA vaccines (0.05; 95% CI, 0.02–0.13) than for the non-replicating viral vector (0.33; 95% CI, 0.22–0.50) and inactivated (0.32; 95% CI, 0.23–0.42) vaccines, with the latter two groups showing similar efficacy. A subgroup analysis of efficacy after single doses of the mRNA and non-replicating viral vector vaccines (no such data were reported for the inactivated vaccine) yielded an overall RR for SARS-COV-2 infection that was 25% higher than the RR for infection after two doses (post single dose: 0.42; 95% CI, 0.20–0.89; post two doses: 0.17; 95% CI, 0.07–0.40), and a significantly higher RR for the mRNA vaccines than for the viral vector vaccines (0.53; 95% CI, 0.23–1.20 vs. 0.29; 95% CI, 0.11–0.76).

**Figure 2:**
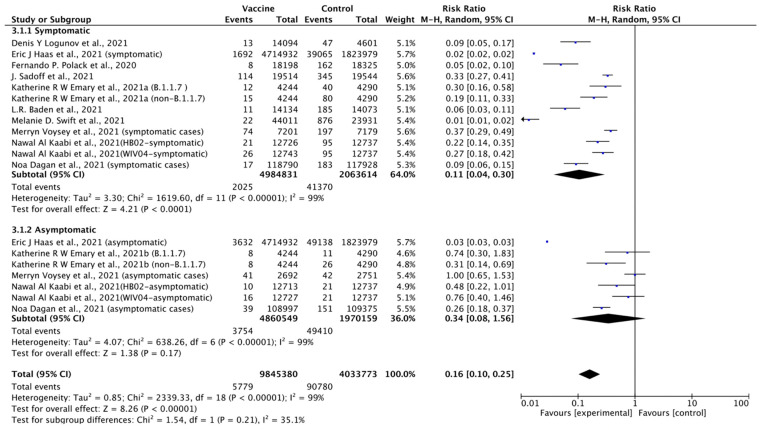
Forest plot of efficacy of vaccines based on different COVID-19 case definitions.

A subgroup analysis of symptomatic and asymptomatic events occurring after two vaccine doses was conducted with pooled data from 10 publications. It showed that the vaccines provided about 84% protection against all SARS-COV-2 infection, 89% protection against symptomatic cases, and 66% protection against asymptomatic cases overall (Figure 2). 

For the meta-analysis of SARS-COV-2 infection severity data, data on severe cases, those requiring hospitalization, and those resulting in death from seven, three, and two publications, respectively, were used. RRs for severe cases, cases requiring hospitalization, and cases resulting in death between the vaccine and control groups were 0.12 (95% CI, 0.05–0.30), 0.08 (95% CI, 0.02–0.24), and 0.14 (95% CI, 0.03–0.69), respectively (Table 5). 

### 3.4. Publication Bias and Risk of Bias Assessment

**Figure 3:**
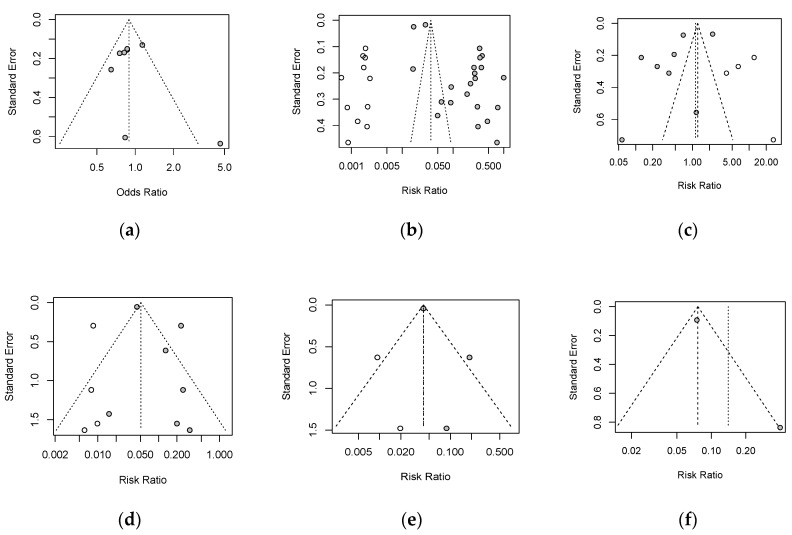
Adjusted funnel plots to examine publication bias: (**a**): safety; (**b**): efficacy after 2 doses; (**c**): efficacy after 1 dose; (**d**): severe cases; (**e**): hospitalization; (**f**): death (Begg’s funnel plot).

Obvious asymmetry was observed in Begg’s funnel plot for safety and efficacy of vaccines, which revealed potential publication bias existing in the selected articles. The trim-and-fill method was used to adjust for funnel plot asymmetry, and the adjusted results are shown in Figure 3, where the results of the pooled analysis are similar to those of the original analysis in terms of safety and efficacy evaluation. 

As described in Table 6, eight articles presented a low risk of bias, whereas four articles were regarded as a moderate risk of bias based on the QUIPS tool assessment. 

## 4. Discussion

This systematic review and meta-analysis were conducted to explore the associations of COVID-19 vaccines with the occurrence of serious adverse events, and to compare the protection provided by different COVID-19 vaccines and the vaccines’ efficacy against different infection types. The analyses provided the best evidence that the current COVID-19 vaccine candidates are not associated directly with serious or life-threatening adverse events. However, the risk ratios for serious adverse events occurring in different organ systems differed among vaccine platforms, suggesting the existence of a relationship between COVID-19 vaccine platforms and serious adverse events, which should prompt further exploration to avoid potential risk for populations with relative diseases. For example, the population with metabolic, musculoskeletal, immune-system, and renal disorders should avoid the receipt of inactivated COVID-19 vaccines, and it is suggested that patients who have vessel diseases should not receive mRNA vaccines. Most reviews and meta-analyses of COVID-19 vaccine safety conducted to date have focused on the risk of adverse events, with less attention paid to the occurrence of serious adverse events related directly to mortality and morbidity. Our analysis of COVID-19 vaccine efficacy was more comprehensive than that of most previous research, which has been limited to the examination of risk ratios for SARS-COV-2 infection after whole-course vaccination. Our findings reveal that all current vaccine platforms provide sufficient protection against SARS-COV-2 infection and substantially decrease the risk of serious infection. mRNA vaccines are most effective against SARS-COV-2 infection. Our findings also suggest that the risk of SARS-COV-2 infection after receipt of the first vaccine dose is almost 2.5-fold greater than that after receipt of the second dose. Given the improved efficacy of the vaccines after the second dose, people should continue to follow protective measures, such as mask wearing and social distancing, after receipt of the first dose. Although the COVID-19 vaccines provide >50% protection against symptomatic and asymptomatic infections, they provide three-fold greater protection against symptomatic than against asymptomatic infections. Thus, measures are required to avoid the risk posed by vaccinated individuals with asymptomatic infections. For example, large-scale screening in high-risk populations may aid the timely detection of asymptomatic cases and reduce the risk of SARS-COV-2 infection spread. 

Another strength of this review is that it synthesized data from phase-3 clinical trials and observational studies based on a strict search strategy, which ensured the acquisition of strong evidence on the topic. However, the review has several limitations. First, the included studies displayed heterogeneity due to differences in study design and sample size. For example, most studies showed that the COVID-19 vaccines protected against SARS-COV-2 infection, with efficacy improving after the second dose. However, the protection provided by viral vector vaccines decreased by 4%. This finding is not consistent with the conclusion drawn by Merryn Voysey et al. [29], who found no difference in vaccine efficacy against asymptomatic cases relative to the control group, possibly due to the inclusion of asymptomatic cases in the analysis and the small sample. Second, the results may be imbalanced, because five of the 12 publications included reported on BNT162b2. Third, visual inspection of the funnel plots revealed asymmetry reflecting publication bias [32]. Thus, the funnel plots were adjusted using the trim-and-fill approach, which did not affect the significance of the outcomes. 

Large-scale vaccination is currently being implemented in many countries and regions to block further spread of SARS-COV-2, and the safety and efficacy of COVID-19 vaccines influence public acceptance and progress toward universal vaccination. With the rapid global mutation of SARS-COV-2, a long-term surveillance system for the safety and efficacy of COVID-19 vaccines is needed, and more research will be required on different vaccine platforms to optimize community vaccination and effectively slow SARS-COV-2 spread.

## 5. Conclusions

This study showed that full COVID-19 vaccination protects most people from SARS-COV-2 infection and reduces the severity of COVID-19. Evidence on the ability of COVID-19 vaccines to cause serious adverse events is insufficient; more data are needed. In addition, data on vaccines developed using other platforms, such as protein subunit vaccines, were not published at the time that this review was conducted, and the articles included in this review exhibited a high degree of heterogeneity. Thus, more research is needed to promote universal vaccination against SARS-COV-2.

## Figures and Tables

**Table 1:** Search criteria on PubMed and EMBASE with PICO framework.

	PubMed	EMBASE
Search terms		
COVID-19 vaccine	“COVID-19 vaccine *”	exp SARS-COV-2 vaccine/or COVID-19 vaccine.mp.
	AND	AND
Endpoints	((Efficacy OR Effectiveness) OR Safety)	((Efficacy.mp. OR Effectiveness.mp.) OR (Safety.mp. or exp Safety/))
Inclusion criteria		
Population	No restrictions on population	
Intervention	COVID-19 vaccines	
Comparison	The population in control group	
Outcomes	Incidence of serious adverse events, and cases infected with COVID-19 after vaccination	

* just means to search on variations of a root word and don’t need to think of all possible term variations but can include them in the search.

**Table 2:** Summary of studies on COVID-19 vaccines included in the systematic review.

Authors, Year [Ref]	Vaccine	Country/ Region	Platform	Population	Study Design	Period	Sample Size	Number of Doses	Route of Administration
L.R. Baden et al., 2021 [19]	mRNA-1273	US	mRNA	≥18 years old	Single-blind, randomized, controlled trial	27 July–23 October 2020	30,420	2	Intramuscular
Merryn Voysey et al., 2020 [20]	AZD1222	UK, Brazil, South Africa	Non-Replicating Viral Vector	≥18 years old	Single-blind, randomized, controlled trial	24 April–4 November 2020	23,848	2	Intramuscular
Denis Y Logunov et al., 2021 [21]	Gam-COVID-Vac	Russia	Non-Replicating Viral Vector	≥18 years old	Double-blind, randomized, controlled trial	7 September–24 November 2020	21,977	2	Intramuscular
Fernando P. Polack et al., 2020 [22]	BNT162b2	US, Argentina, Brazil, South Africa	mRNA	≥16 years old	Single-blind, randomized, controlled trial	27 July–14 November 2020	44,820	2	Intramuscular
Katherine R W Emary et al., 2021 [23]	AZD1222	UK	Non-Replicating Viral Vector	≥18 years old	Single-blind, randomized, controlled trial	31 May–13 November 2020	8534	2	Intramuscular
J. Sadoff et al., 2021 [24]	Ad26.COV2.S	Argentina, Brazil, Chile, Colombia, Mexico, Peru South Africa, US	Non-Replicating Viral Vector	≥18 years old	Double-blind, randomized, controlled trial	21 September 2020–22 January 2021	44,325	1	Intramuscular
Eric J Haas et al., 2021 [25]	BNT162b2	Israel	mRNA	≥16 years old	Observational study	24 January–3 April, 2021	6,538,911	2	Intramuscular
Noa Dagan et al., 2021 [26]	BNT162b2	Israel	mRNA	≥16 years old healthcare workers	Observational study	20 December 2020–1 February 2021	769,958	2	Intramuscular
Nick K Jones et al., 2021 [27]	BNT162b2	UK	mRNA	Healthcare workers	Observational study	18 January–31 January 2021	8819	1	Intramuscular
Melanie D. Swift et al., 2021 [28]	BNT162b2	US	mRNA	Healthcare workers	Observational study	1 January–31 March 2021	76,000	2	Intramuscular
Merryn Voysey et al., 2021 [29]	AZD1222	UK, Brazil, South Africa	Non-Replicating Viral Vector	≥18 years old	Randomized, controlled trial	23 April–6 December 2020	24,422	2	Intramuscular
Nawal Al Kaabi Et al., 2021 [30]	BBIBP-CorV	UAE, Bahrain	Inactivated	≥18 years old	Double-blind, randomized, controlled trial	16 July–31 December 2020	40,382	2	Intramuscular

**Table 3:** The summary of safety and efficacy for 3 different vaccine platforms.

	mRNA	Non-Replicating Viral Vector	Inactivated	Overall (95% CI)
**Safety ^1^** (Number of participants with serious adverse events/total number of participants in the group)				0.86 (0.7, 1.06)
Vaccine Group ^#^	145/51,466	194/50,343	123/26,935	
Control Group ^#^	120/51,352	210/38,957	156/26,906	
Odds ratio (95% CI)	1.47 (0.65, 3.29)	0.76 (0.62, 0.93)	0.79 (0.62, 1.00)	
**Efficacy after 2 doses ^2^** (Number of cases/total number of participants in the group)				0.17 (0.07, 0.40)
Vaccine Group *	6371/5,019,062	285/60,477	73/25,440	
Control Group *	111,554/2,107,611	788/51,235	232/25,444	
Risk ratio (95% CI)	0.05 (0.02, 0.13)	0.33 (0.22, 0.50)	0.32 (0.23, 0.42)	
**Efficacy after 1 dose ^2^** (Number of cases/total number of participants in the group)				0.42 (0.20, 0.89)
Vaccine Group *	1077/603,427	54/27,492	/	
Control Group *	904/601,411	156/17,426	/	
Risk ratio (95% CI)	0.53 (0.23, 1.20)	0.29 (0.11, 0.76)	/	

^1^ Measured using odds ratios for serious adverse events. ^2^ Evaluated using risk ratios of confirmed cases after vaccination. ^#^ The number of people with serious adverse events/The total number of people in the vaccine group (control group). * The number of confirmed cases/The total number of people in the vaccine group (control group).

**Table 4:** Proportions of serious adverse events by system organ class ^1^.

Overall	Inactivated		Non-Replicating Viral Vector			mRNA		SAEs ^2^
	HB02	WIV04	AZD1222	Gam-COVID-Vac	Ad26. CoV2.S	mRNA-1273	BNT162b2	
0.01	0.19	0.14	0.00	0.00	0.00	0.00	0.00	Nutrition and metabolism disorders
(0.00, 0.08)	(0.10, 0.31)	(0.07, 0.25)	(0.00, 0.04)	(0.00, 0.08)	(0.00, 0.04)	(0.00, 0.02)	(0.00, 0.60)	
0.05	0.12	0.13	0.10	0.09	0.00	0.02	0.00	Gastrointestinal disorders
(0.01, 0.10)	(0.05, 0.23)	(0.06, 0.23)	(0.04, 0.18)	(0.02, 0.20)	(0.00, 0.04)	(0.01, 0.06)	(0.00, 0.60)	
0.04	0.09	0.13	0.06	0.02	0.02	0.01	0.00	Musculoskeletal and connective tissue disorders
(0.01, 0.08)	(0.03, 0.19)	(0.06, 0.23)	(0.02, 0.13)	(0.00, 0.11)	(0.00, 0.08)	(0.00, 0.03)	(0.00, 0.60)	
0.03	0.17	0.08	0.05	0.04	0.00	0.00	0.00	Renal and urinary disorders
(0.00, 0.08)	(0.08, 0.29)	(0.03, 0.17)	(0.01, 0.12)	(0.01, 0.15)	(0.00, 0.04)	(0.00, 0.03)	(0.00, 0.60)	
0.05	0.08	0.05	0.08	0.04	0.02	0.01	0.25	Nervous system disorders
(0.03, 0.10)	(0.03, 0.19)	(0.01, 0.13)	(0.03 0.16)	(0.01, 0.15)	(0.00, 0.08)	(0.00, 0.03)	(0.01, 0.81)	
0.05	0.15	0.00	0.08	0.13	0.01	0.07	0.25	Cardiac disorders
(0.01, 0.11)	(0.07, 0.27)	(0.00, 0.06)	(0.03, 0.16)	(0.05, 0.16)	(0.00, 0.06)	(0.04, 0.12)	(0.01, 0.81)	
0.01	0.02	0.06	0.01	0.00	0.00	0.02	0.00	Respiratory system disorders
(0.00, 0.02)	(0.00, 0.09)	(0.02, 0.15)	(0.00, 0.06)	(0.00, 0.08)	(0.00, 0.04)	(0.01, 0.05)	(0.00, 0.60)	
0.01	0.08	0.06	0.00	0.02	0.01	0.02	0.00	Blood and immune system disorders
(0.00, 0.04)	(0.03, 0.19)	(0.02, 0.15)	(0.00, 0.04)	(0.00, 0.11)	(0.00, 0.06)	(0.01, 0.05)	(0.00, 0.60)	
0.02	0.03	0.05	0.01	0.02	0.01	0.04	0.00	General or administration site conditions
(0.01, 0.04)	(0.00, 0.12)	(0.01, 0.13)	(0.00, 0.06)	(0.00, 0.11)	(0.00, 0.06)	(0.02, 0.08)	(0.00, 0.60)	
0.05	0.05	0.02	0.00	0.21	0.00	0.08	0.5	Vessels and lymphatic vessels disorders
(0.01, 0.12)	(0.01, 0.14)	(0.00, 0.08)	(0.00, 0.04)	(0.11, 0.36)	(0.00, 0.04)	(0.04, 0.12)	(0.07, 0.93)	
0.00	0.03	0.00	0.00	0.06	0.00	0.01	0.00	Hepatobiliary system disorders
(0.00, 0.01)	(0.00, 0.12)	(0.00, 0.06)	(0.00, 0.04)	(0.01, 0.18)	(0.00, 0.04)	(0.00, 0.04)	(0.00, 0.60)	
0.00	0.02	0.02	0.01	0.00	0.00	0.00	0.00	Skin and subcutaneous tissue disorders
(0.00, 0.001)	(0.00, 0.09)	(0.00, 0.08)	(0.00, 0.06)	(0.00, 0.08)	(0.00, 0.04)	(0.00, 0.02)	(0.00, 0.60)	
0.00	0.00	0.00	0.08	0.06	0.00	0.00	0.00	Reproductive system and breast disorders
(0.00, 0.03)	(0.00, 0.06)	(0.00, 0.60)	(0.03, 0.16)	(0.01, 0.18)	(0.00, 0.04)	(0.00, 0.02)	(0.00, 0.60)	
0.13	0.34	0.33	0.21	0.17	0.00	0.04	0.00	Infections and infestations
(0.02, 0.28)	(0.22, 0.47)	(0.22, 0.47)	(0.13, 0.32)	(0.08, 0.31)	(0.00, 0.04)	(0.02, 0.07)	(0.00, 0.60)	
0.07	0.19	0.13	0.12	0.13	0.00	0.03	0.25	Injury, poisoning and procedural complications
(0.02, 0.16)	(0.10, 0.31)	(0.06, 0.23)	(0.06, 0.21)	(0.05, 0.26)	(0.00, 0.04)	(0.01, 0.07)	(0.01, 0.81)	

^1^ This table lists common serious system disorders and corresponding proportions in different vaccines with 95% CI; the proportion of different SAEs was calculated as the number of participants with specific SAE/the total number of participants with SAEs; ^2^ SAEs: serious adverse events.

**Table 5:** The overall comparison of severity of COVID-19 in vaccine and control group.

	Vaccine Group		Control Group		Risk Ratio (95% CI)
	Events (N)	Total (N)	Events (N)	Total (N)	
Severe cases	382	4,917,815	3325	2,026,409	0.12 (0.05, 0.30)
Hospitalized cases	599	4,837,007	5547	1,945,570	0.08 (0.02, 0.24)
Death	140	4,825,033	720	1,933,987	0.14 (0.03, 0.69)

**Table 6:** Risk of bias assessment based on Quality in Prognostic Studies (QUIPS) tool (low = “low risk”, middle = “middle risk”, high = “high risk”).

Biases	Study Population	Study Attrition	Prognostic Factor Measurement	Outcome Measurement	Study Confounding	Statistical Analysis and Reporting	Overall
L.R. Baden et al., 2021 [19]	low	high	low	low	low	low	low
Merryn Voysey et al., 2020 [20]	low	high	low	low	low	low	low
Denis Y Logunov et al., 2021 [21]	low	high	low	low	low	low	low
Fernando P. Polack et al., 2020 [22]	low	high	low	low	low	low	low
Katherine R W Emary et al., 2021 [23]	low	high	low	low	high	low	low
J. Sadoff et al., 2021 [24]	low	high	low	low	low	low	low
Eric J Haas et al., 2021 [25]	middle	high	low	low	high	low	middle
Noa Dagan et al., 2021 [26]	low	low	low	low	low	low	low
Nick K Jones et al., 2021 [27]	low	high	low	low	high	middle	middle
Melanie D. Swift et al., 2021 [28]	middle	high	low	low	high	low	middle
Merryn Voysey et al., 2021 [29]	low	high	low	low	high	middle	middle
Nawal Al Kaabi Et al., 2021 [30]	low	middle	low	low	middle	low	low

## Supplementary Materials

The following are available online at www.mdpi.com/article/10.3390/vaccines9090989/s1, Figure S1. Forest plot of association between serious adverse events and COVID-19 vaccine, Figure S2. Forest plot of association between COVID-19 cases and vaccination after the 2 dose, Figure S3. Forest plot of association between COVID-19 cases and vaccination after the first dose, Figure S4. Forest plot of association between severity of COVID-19 and vaccines. (a): Severe cases; (b): Hospitalized cases; (c) Death.

## References

[1] Petrosillo N., Viceconte G., Ergonul O., Ippolito G., Petersen E. (2020). COVID-19, SARS and MERS: Are they closely related?. Clin. Microbiol. Infect..

[2] Qin C., Liu F., Yen T.-C., Lan X. (2020). 18F-FDG PET/CT findings of COVID-19: A series of four highly suspected cases. Eur. J. Nucl. Med. Mol. Imaging.

[3] World Health Organization (2021). WHO Coronavirus (COVID-19) Dashboard. https://covid19.who.int/.

[4] Wilder-Smith A., Freedman D.O. (2020). Isolation, quarantine, social distancing and community containment: Pivotal role for old-style public health measures in the novel coronavirus (2019-nCoV) outbreak. J. Travel Med..

[5] Zhang Y., Zeng G., Pan H., Li C., Hu Y., Chu K., Han W., Chen Z., Tang R., Yin W. (2021). Safety, tolerability, and immunogenicity of an inactivated SARS-CoV-2 vaccine in healthy adults aged 18–59 years: A randomised, double-blind, placebo-controlled, phase 1/2 clinical trial. Lancet Infect. Dis..

[6] Xia S., Duan K., Zhang Y., Zhao D., Zhang H., Xie Z., Li X., Peng C., Zhang Y., Zhang W. (2020). Effect of an Inactivated Vaccine Against SARS-CoV-2 on Safety and Immunogenicity Outcomes: Interim Analysis of 2 Randomized Clinical Trials. JAMA.

[7] Gao Q., Bao L., Mao H., Wang L., Xu K., Yang M., Li Y., Zhu L., Wang N., Lv Z. (2020). Development of an inactivated vaccine candidate for SARS-CoV-2. Science.

[8] Palacios R., Batista A.P., Albuquerque C.S.N., Patiño E.G., Santos J.D.P., Tilli Reis Pessoa Conde M., Piorelli R.D.O., Pereira Júnior L.C., Raboni S.M., Ramos F. (2021). Efficacy and Safety of a COVID-19 Inactivated Vaccine in Healthcare Professionals in Brazil: The PROFISCOV Study. SSRN.

[9] Feng L., Wang Q., Shan C., Yang C., Feng Y., Wu J., Liu X., Zhou Y., Jiang R., Hu P. (2020). An adenovirus-vectored COVID-19 vaccine confers protection from SARS-COV-2 challenge in rhesus macaques. Nat. Commun..

[10] Zhu F.-C., Guan X.-H., Li Y.-H., Huang J.-Y., Jiang T., Hou L.-H., Li J.-X., Yang B.-F., Wang L., Wang W.-J. (2020). Immunogenicity and safety of a recombinant adenovirus type-5-vectored COVID-19 vaccine in healthy adults aged 18 years or older: A randomised, double-blind, placebo-controlled, phase 2 trial. Lancet.

[11] Alberer M., Gnad-Vogt U., Hong H.S., Mehr K.T., Backert L., Finak G., Gottardo R., Bica M.A., Garofano A., Koch S.D. (2017). Safety and immunogenicity of a mRNA rabies vaccine in healthy adults: An open-label, non-randomised, prospective, first-in-human phase 1 clinical trial. Lancet.

[12] World Health Organization (2021). What Is COVID-19 Vaccine Efficacy?. https://www.afro.who.int/news/what-covid-19-vaccine-efficacy.

[13] Li J., Ulitzky L., Silberstein E., Taylor D.R., Viscidi R. (2013). Immunogenicity and Protection Efficacy of Monomeric and Trimeric Recombinant SARS Coronavirus Spike Protein Subunit Vaccine Candidates. Viral Immunol..

[14] Keech C., Albert G., Cho I., Robertson A., Reed P., Neal S., Plested J.S., Zhu M., Cloney-Clark S., Zhou H. (2020). Phase 1–2 Trial of a SARS-CoV-2 Recombinant Spike Protein Nanoparticle Vaccine. N. Engl. J. Med..

[15] Yang S., Li Y., Dai L., Wang J., He P., Li C., Fang X., Wang C., Zhao X., Huang E. (2021). Safety and immunogenicity of a recombinant tandem-repeat dimeric RBD-based protein subunit vaccine (ZF2001) against COVID-19 in adults: Two randomised, double-blind, placebo-controlled, phase 1 and 2 trials. Lancet Infect. Dis..

[16] Moher D., Liberati A., Tetzlaff J., Altman D.G., The PRISMA Group (2009). Preferred Reporting Items for Systematic Reviews and Meta-Analyses: The PRISMA Statement. PLoS Med..

[17] Hayden J.A., Van Der Windt D.A., Cartwright J.L., Côté P., Bombardier C. (2013). Assessing Bias in Studies of Prognostic Factors. Ann. Intern. Med..

[18] MedDRA (2019). Introductory Guide for Standardised MedDRA Queries (SMQs) Version 21.0.

[19] Baden L.R., El Sahly H.M., Essink B., Kotloff K., Frey S., Novak R., Diemert D., Spector S.A., Rouphael N., Creech C.B. (2021). Efficacy and Safety of the mRNA-1273 SARS-CoV-2 Vaccine. N. Engl. J. Med..

[20] Voysey M., Clemens S.A.C., Madhi S.A., Weckx L.Y., Folegatti P.M., Aley P.K., Angus B., Baillie V.L., Barnabas S.L., Bhorat Q.E. (2021). Safety and efficacy of the ChAdOx1 nCoV-19 vaccine (AZD1222) against SARS-CoV-2: An interim analysis of four randomised controlled trials in Brazil, South Africa, and the UK. Lancet.

[21] Logunov D.Y., Dolzhikova I.V., Shcheblyakov D.V., Tukhvatulin A.I., Zubkova O.V., Dzharullaeva A.S., Kovyrshina A.V., Lubenets N.L., Grousova D.M., Erokhova A.S. (2021). Safety and efficacy of an rAd26 and rAd5 vector-based heterologous prime-boost COVID-19 vaccine: An interim analysis of a randomised controlled phase 3 trial in Russia. Lancet.

[22] Polack F.P., Thomas S.J., Kitchin N., Absalon J., Gurtman A., Lockhart S., Perez J.L., Marc G.P., Moreira E.D., Zerbini C. (2020). Safety and Efficacy of the BNT162b2 mRNA Covid-19 Vaccine. N. Engl. J. Med..

[23] Emary K.R.W., Golubchik T., Aley P.K., Ariani C.V., Angus B., Bibi S., Blane B., Bonsall D., Cicconi P., Charlton S. (2021). Efficacy of ChAdOx1 nCoV-19 (AZD1222) vaccine against SARS-CoV-2 variant of concern 202012/01 (B.1.1.7): An exploratory analysis of a randomised controlled trial. Lancet.

[24] Sadoff J., Gray G., Vandebosch A., Cárdenas V., Shukarev G., Grinsztejn B., Goepfert P.A., Truyers C., Fennema H., Spiessens B. (2021). Safety and Efficacy of Single-Dose Ad26.COV2.S Vaccine against Covid-19. N. Engl. J. Med..

[25] Haas E.J., Angulo F.J., McLaughlin J.M., Anis E., Singer S.R., Khan F., Brooks N., Smaja M., Mircus G., Pan K. (2021). Impact and effectiveness of mRNA BNT162b2 vaccine against SARS-CoV-2 infections and COVID-19 cases, hospitalisations, and deaths following a nationwide vaccination campaign in Israel: An observational study using national surveillance data. Lancet.

[26] Dagan N., Barda N., Kepten E., Miron O., Perchik S., Katz M.A., Hernán M.A., Lipsitch M., Reis B., Balicer R.D. (2021). BNT162b2 mRNA Covid-19 Vaccine in a Nationwide Mass Vaccination Setting. N. Engl. J. Med..

[27] Jones N.K., Rivett L., Seaman S., Samworth R.J., Warne B., Workman C., Ferris M., Wright J., Quinnell N., Shaw A. (2021). Single-dose BNT162b2 vaccine protects against asymptomatic SARS-CoV-2 infection. eLife.

[28] Swift M.D., Breeher L.E., Tande A.J., Tommaso C.P., Hainy C.M., Chu H., Murad M.H., Berbari E.F., Virk A. (2021). Effectiveness of Messenger RNA Coronavirus Disease 2019 (COVID-19) Vaccines Against Severe Acute Respiratory Syndrome Coronavirus 2 (SARS-CoV-2) Infection in a Cohort of Healthcare Personnel. Clin. Infect. Dis..

[29] Voysey M., Clemens S.A.C., Madhi S.A., Weckx L.Y., Folegatti P.M., Aley P.K., Angus B., Baillie V.L., Barnabas S.L., Bhorat Q.E. (2021). Single-dose administration and the influence of the timing of the booster dose on immunogenicity and efficacy of ChAdOx1 nCoV-19 (AZD1222) vaccine: A pooled analysis of four randomised trials. Lancet.

[30] Al Kaabi N., Zhang Y., Xia S., Yang Y., Al Qahtani M.M., Abdulrazzaq N., Al Nusair M., Hassany M., Jawad J.S., Abdalla J. (2021). Effect of 2 Inactivated SARS-CoV-2 Vaccines on Symptomatic COVID-19 Infection in Adults: A Randomized Clinical Trial. JAMA.

[31] US Food and Drug Administration (2007). Guidance for Industry: Toxicity Grading Scale for Healthy Adult and Adolescent Volunteers Enrolled in Preventive Vaccine Clinical Trials.

[32] Higgins J.P., Thomas J., Chandler J., Cumpston M., Li T., Page M.J., Welch V.A. (2019). Cochrane Handbook for Systematic Reviews of Interventions.

