# Supplementary material for: Safety and Efficacy of COVID-19 Vaccines: A Systematic Review and Meta-Analysis of Different Vaccines at Phase 3
Source: Vaccines (Basel). 2021 Sep 4;9(9):989. doi: 10.3390/vaccines9090989 (PMC8473448; doi:10.3390/vaccines9090989)
Supplement: Supplementary file 1 [file vaccines-09-00989-s001.zip › vaccines-1332506-supplementary.pdf]

Supplementary

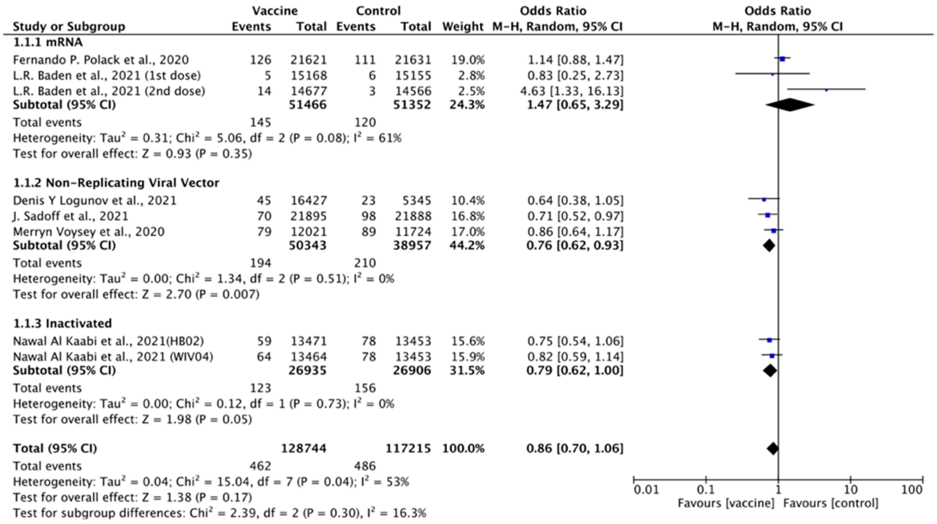

Figure S1. Forest plot of association between serious adverse events and COVID-19 vaccine.

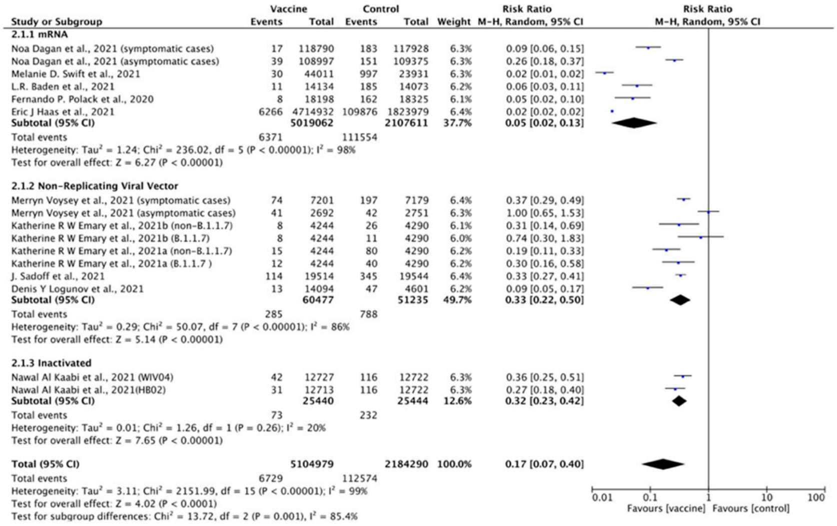

Figure S2. Forest plot of association between COVID-19 cases and vaccination after the 2 dose.

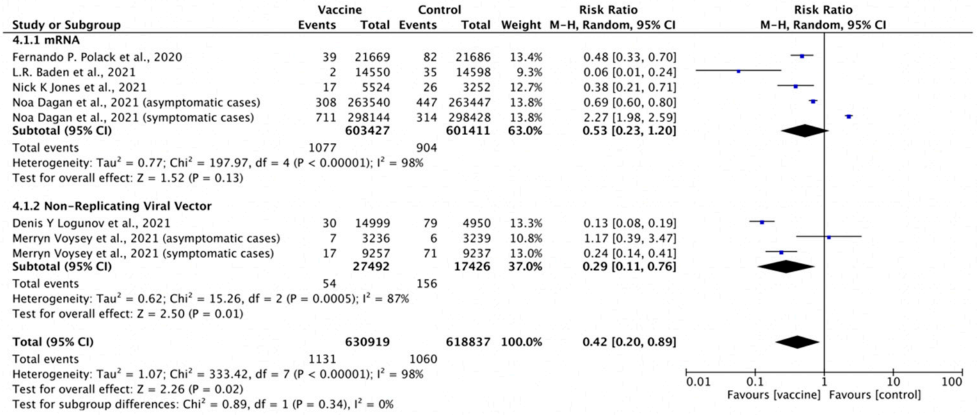

Figure S3. Forest plot of association between COVID-19 cases and vaccination after the first dose.

a.

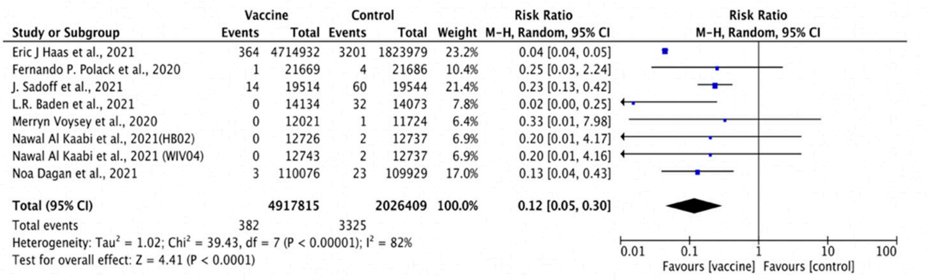

b.

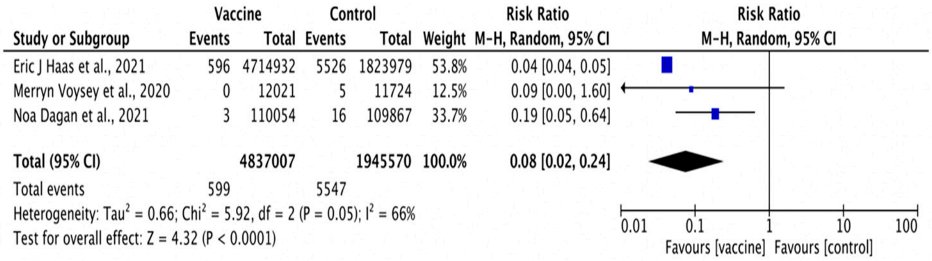

c.

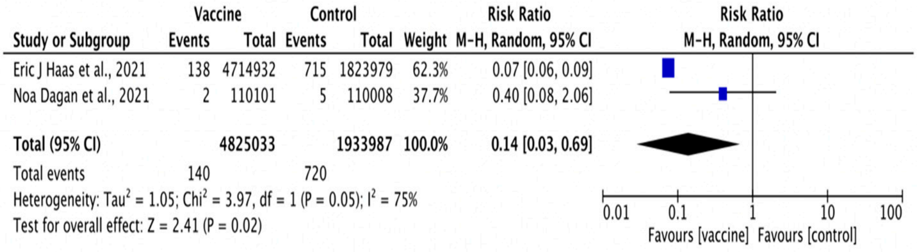

Figure S4. Forest plot of association between severity of COVID-19 and vaccines. (a): Severe cases; (b): Hospitalized cases; (c): Death.
